# The Moss *Leptodictyum riparium* Counteracts Severe Cadmium Stress by Activation of Glutathione Transferase and Phytochelatin Synthase, but Slightly by Phytochelatins

**DOI:** 10.3390/ijms21051583

**Published:** 2020-02-26

**Authors:** Erika Bellini, Viviana Maresca, Camilla Betti, Monica Ruffini Castiglione, Debora Fontanini, Antonella Capocchi, Carlo Sorce, Marco Borsò, Laura Bruno, Sergio Sorbo, Adriana Basile, Luigi Sanità di Toppi

**Affiliations:** 1 Department of Biology, University of Pisa, 56126 Pisa, Italy; erika.bellini@biologia.unipi.it (E.B.); monica.ruffini.castiglione@unipi.it (M.R.C.); debora.fontanini@unipi.it (D.F.); antonella.capocchi@unipi.it (A.C.); carlo.sorce@unipi.it (C.S.); 2 Department of Biology, University of Rome “Tor Vergata”, 00133 Rome, Italy; laura.bruno@uniroma2.it; 3 Department of Biology, University of Naples “Federico II”, 80138 Naples, Italy; viviana.maresca@unina.it (V.M.); adriana.basile@unina.it (A.B.); 4 Department of Medicine, University of Perugia, 06123 Perugia, Italy; camilla.betti@unipg.it; 5 Department of Surgery, Medical, Molecular, and Critical Area Pathology, University of Pisa, 56124 Pisa, Italy; marco.borso@student.unisi.it; 6 Centro di Servizi Metrologici Avanzati (CeSMA), Microscopy Section, University of Naples “Federico II”, 80126 Naples, Italy; sersorbo@unina.it

**Keywords:** bryophytes, cadmium, γ-glutamylcysteine, glutathione, metals, *Leptodictyum riparium*, monochlorobimane, phytochelatins, ROS

## Abstract

In the present work, we investigated the response to Cd in *Leptodictyum riparium*, a cosmopolitan moss (Bryophyta) that can accumulate higher amounts of metals than other plants, even angiosperms, with absence or slight apparent damage. High-performance liquid chromatography followed by electrospray ionization tandem mass spectrometry of extracts from *L. riparium* gametophytes, exposed to 0, 36 and 360 µM Cd for 7 days, revealed the presence of γ-glutamylcysteine (γ-EC), reduced glutathione (GSH), and traces of phytochelatins. The increase in Cd concentrations progressively augmented reactive oxygen species levels, with activation of both antioxidant (catalase and superoxide dismutase) and detoxifying (glutathione-*S-*transferase) enzymes. After Cd treatment, cytosolic and vacuolar localization of thiol peptides was performed by means of the fluorescent dye monochlorobimane and subsequent observation with confocal laser scanning microscopy. The cytosolic fluorescence observed with the highest Cd concentrations was also consistent with the formation of γ-EC-bimane in the cytosol, possibly catalyzed by the peptidase activity of the *L. riparium* phytochelatin synthase. On the whole, activation of phytochelatin synthase and glutathione-*S*-transferase, but minimally phytochelatin synthesis, play a role to counteract Cd toxicity in *L. riparium*, in this manner minimizing the cellular damage caused by the metal. This study strengthens previous investigations on the *L. riparium* ability to efficiently hinder metal pollution, hinting at a potential use for biomonitoring and phytoremediation purposes.

## 1. Introduction

Trace metals, such as Cd, Hg, Pb, Cr(VI), etc., are important environmental pollutants, particularly in areas characterized by a strong anthropogenic pressure [1]. Their presence in the atmosphere, soil, and water, even at extremely low concentrations, can seriously damage all living organisms. Specifically, Cd is a widespread metal that is released into the environment by power stations, heating systems, electroplating, smelting, urban traffic, cement factories, and, sometimes, as a byproduct of some fertilizers [1]. By possessing a toxicity from 2- to 20-fold higher than many other metals, Cd is very harmful to a large number of organisms [2].

In plant cells, Cd ions are highly noxious even at low concentrations, with subsequent severe negative effects [3]. Unlike other metals, Cd does not directly induce oxidative stress [4,5] via Fenton and/or Haber-Weiss reactions [6], but rather disturbs the overall cellular redox balance and, consequently, affects the reactive oxygen species (ROS) levels [7]. In fact, Cd toxicity mainly originates from non-functional binding to various ligands that are meant to bind other divalent metals, i.e., Zn. Less known, a ligand may also be chlorophyll, where Cd^2+^ replaces Mg^2+^ as the central ion. Therefore, although not redox active, Cd exposure leads to enhanced production of ROS. Another reason is that Cd exposure reduces the capability of scavenging ROS [8]. In this regard, Cd can activate or even inhibit several antioxidant enzymes, such as superoxide dismutase (SOD; EC 1.15.1.1), which catalyzes the production of O_2_ and H_2_O_2_ from the radical anion superoxide (•O_2_^–^); catalase (CAT; EC 1.11.1.6), which decomposes H_2_O_2_ into O_2_ and H_2_O, and many others. Among these enzymes, the multifunctional enzyme glutathione-*S*-transferase (GST; EC 2.5.1.18) [9] can simultaneously counteract oxidative stress by enhancing ROS quenching, and detoxify a number of electrophilic xenobiotics or chemical elements, including Cd, both in yeast [10] and in plants [11,12,13]. Particularly, GST catalyzes an intracellular detoxification reaction of metals or noxious compounds by forming first a cytosolic conjugate between the thiol peptide glutathione (GSH) and the toxic element/substance, followed by sequestering this conjugate (GS conjugate) into the vacuolar compartment of the plant cell [14] by means of ATP binding cassette (ABC) transporters [15]. To detect xenobiotic or metal detoxification by conjugation, and the subsequent translocation of the conjugate to various compartments, the dye monochlorobimane (MCB) can be useful, because it becomes fluorescent after conjugation to GSH and, to a lesser extent, to other thiol peptides [16,17]. Time course experiments with MCB can be monitored by confocal laser scanning microscopy (CLSM).

Besides, in higher plants, an important metal detoxification system is based on the so-called phytochelatins (PCn) [1], directly derived from GSH. PCn are thiol peptide compounds with the general structure (γ-glutamylcysteine [EC])_n_-glycine, with n usually ranging from 2 to 5. Due to the cysteine thiol groups, PCn chelate Cd or other metals and compartmentalize them in the vacuole [18], in order to quickly detoxify the cytosolic environment. From a biosynthetic point of view, PCn are synthetized from GSH by the activation of the enzyme phytochelatin synthase (PCS), a γ-EC dipeptidyl (trans)peptidase (EC 2.3.2.15) that is constitutively expressed in the plant cytosol [19]. PCS activation is self-regulated, because its products (that is, PCn) chelate Cd, and the reaction stops when free Cd ions are no longer available [20]. However, other than being a γ-EC transpeptidase, PCS is also a cysteine peptidase that may regulate the cytosolic catabolism of GS-conjugates [21,22,23]. In this case, GS-conjugates with MCB (GS-bimane) can be cleaved into γ-EC and glycine, a reaction stimulated by some metals, particularly Cd, Zn, and Cu [21,23].

So far, the vast majority of studies on responses to metals (in particular Cd) in plants have been performed in higher plants, especially angiosperms, whereas only few aspects have been thoroughly investigated in bryophytes, considered the earliest-diverged lineages of land plants [24]. Due to their ancientness and their peculiar phylogenetic position [25,26], bryophytes (liverworts, mosses, and hornworts) are pivotal for reconstructing the origin of morphofunctional, ultrastructural, and cytohistological traits of plants in the transition from water to land, including those related to metal detoxification and homeostasis [24,27,28]. Moreover, bryophytes possess a very high surface/volume ratio, have an elevated cation exchange capacity, do not develop strong hydrophobic barriers, and, consequently, are prone to the absorption of (metal) contaminants from all environmental matrices. For these reasons, bryophytes are considered extraordinary systems for the monitoring of pollution and, more particularly, of metal contamination [29].

Previous studies have demonstrated that the cosmopolitan moss *Leptodictyum riparium* (Bryophyta) can accumulate, and seemingly tolerate, very high concentrations of toxic metals, including Cd [30,31,32], with a bioconcentration factor higher than that of other plants, even of some angiosperms [33]. Thanks to its apparent tolerance to metal stress and to its high efficiency for metal removal, *L. riparium* has therefore been proposed as a useful tool for biomonitoring metal contamination, as well as for carrying out phytoremediation projects in polluted areas [31,32,33]. Interestingly, *L. riparium* performs little Cd immobilization at the cell wall level, and therefore the metal enters the cytosol rather easily [31,34]. Thus, the apparent Cd tolerance showed by this moss in the open environment might be due to efficient intracellular (symplastic), rather than to cell wall (apoplastic), detoxification processes.

Although the *L. riparium* gametophytes collected in the open appear to possess an elevated tolerance to Cd [31,32], until now no *ad hoc* studies have been carried out in the laboratory-confined environment. The latter experiments could therefore address the issue in mechanistic terms, when the moss is subjected to strong and prolonged Cd stress in controlled conditions. Thus, in this work, we hypothesize that the high ability of *L. riparium* gametophytes to effectively counteract Cd stress could rely on the activation of intracellular responses based on some antioxidant/detoxifying enzymes, such as SOD, CAT, and GST, as well as on the presence of thiol-peptide compounds, particularly γ-EC, GSH, and PCn. An in-depth observation of Cd effects in this moss is here provided by CLSM imaging of MCB-stained thiols, and by optical/electron microscopy techniques. The overall results can be useful to understand the basis of the complex response mechanisms carried out by mosses and other early land plants when exposed, even outdoors, to severe metal stress.

## 2. Results

### 2.1. ROS Production and Antioxidant Response to Cd

**Figure 1:**
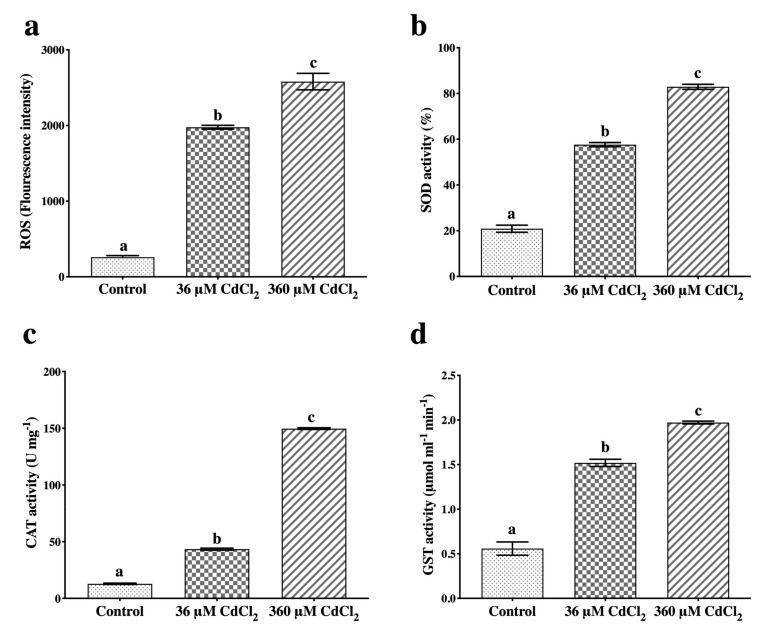
ROS amount and antioxidant/detoxifying enzyme activities in *L. riparium* gametophytes treated with 0 (Control), 36 or 360 μM CdCl_2_ for 7 days. (**a**) ROS production; activities of (**b**) superoxide dismutase, SOD; (**c**) catalase, CAT; (**d**) glutathione-*S*-transferase, GST. Values are mean ± SE; bars not accompanied by the same letter are significantly different at *p* < 0.05.

In Cd-treated gametophytes, the amount of ROS highly increased compared to controls (Figure 1a), and the antioxidant/detoxifying enzymes under investigation were also progressively activated by the two Cd concentrations. Actually, the SOD activity in 360-μM-treated samples was 20% and 60% higher than under the 36 μM treatment and the control, respectively (Figure 1b), whereas CAT increased up to 150 U/mg for 360 μM-treated samples (Figure 1c). Both Cd concentrations markedly enhanced the GST activity, reaching a value of 2.0 μmol mL^−1^ min^−1^ in the 360-μM-treated gametophytes (Figure 1d). Data are given in detail in Table S1.

### 2.2. L. riparium Possesses a Functional PCS that Produces Cd-Induced PCn in Vitro and in Vivo

**Figure 2:**
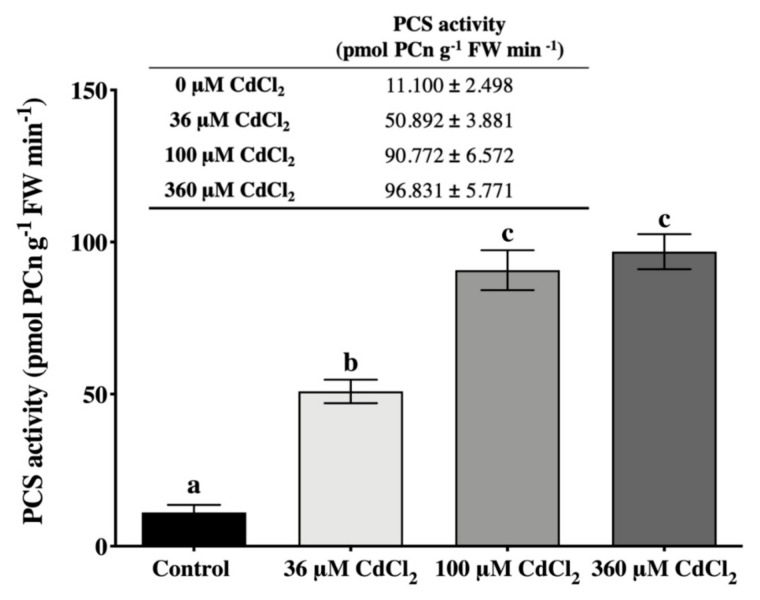
*In vitro* PCS activity of *L. riparium* gametophytes incubated with 0 (Control), 36, 100, and 360 μM CdCl_2_ for 90 min. Values are mean ± SE; bars not accompanied by the same letter are significantly different at *p* < 0.05.

The *in vitro* assay of the PCS from *L. riparium* gametophytes clearly revealed that the enzyme was activated already at the lowest Cd concentration supplied (36 μM). The increase in Cd concentration up to 100 μM led to an enhanced PCS activation, followed by a *plateau* state of the activity at the highest Cd concentration (360 μM) (Figure 2).

**Figure 3:**
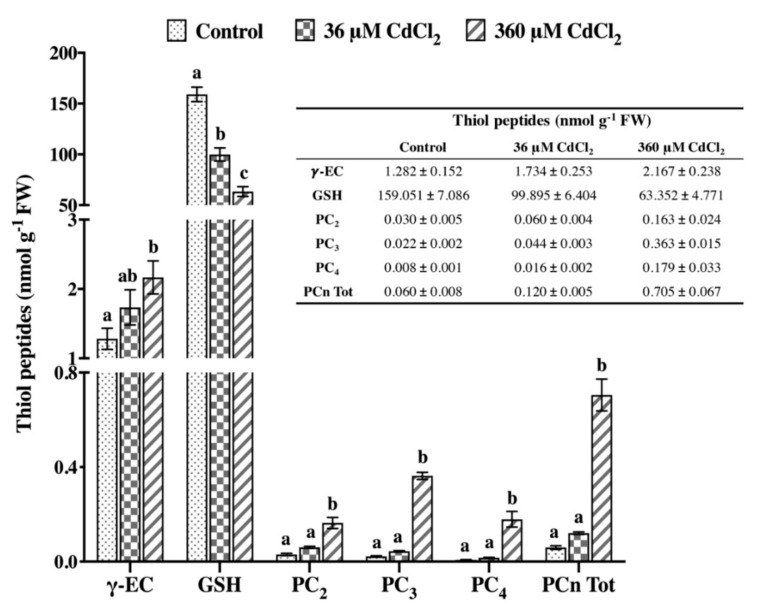
Content of γ-EC, GSH and PCn in *L. riparium* gametophytes, exposed to 0 (Control), 36 and 360 μM CdCl_2_ for 7 days. Values are mean ± SE; within each group of thiol peptides, bars not accompanied by the same letter are significantly different at *p* < 0.05.

Both in control and in Cd-treated samples, the ability of *L. riparium* gametophytes to synthesize thiol peptides *in vivo* was here demonstrated; in particular, the presence of γ-EC, GSH, and PC_2–4_ was distinctly detected (Figure 3). The amount of PCn, although at trace levels, significantly increased only with the 360 µM CdCl_2_ treatment, and not with 36 µM CdCl_2_, as compared to controls. The highest Cd concentration led to an increased synthesis of all PCn oligomers (PC_2_, PC_3_, and PC_4_) (Figure 3). Differently, the GSH levels progressively decreased with the increase in Cd concentrations (Figure 3), and the γ-EC levels showed an upward trend between the controls and the 36-µM-treated samples, whereas the difference was significant between the control and the 360 µM CdCl_2_-treated samples (Figure 3).

The presence of PC_2_, PC_3_, and PC_4_ oligomers was evident in the chromatograms obtained by high-performance liquid chromatography-electrospray ionization tandem mass spectrometry (HPLC-ESI-MS-MS) of extracts from *L. riparium* gametophytes, exposed to Cd for 7 days (see Figure S1 for some exemplifying chromatograms).

### 2.3. Confocal Imaging of MCB Staining and Chlorophyll Autofluorescence

**Figure 4:**
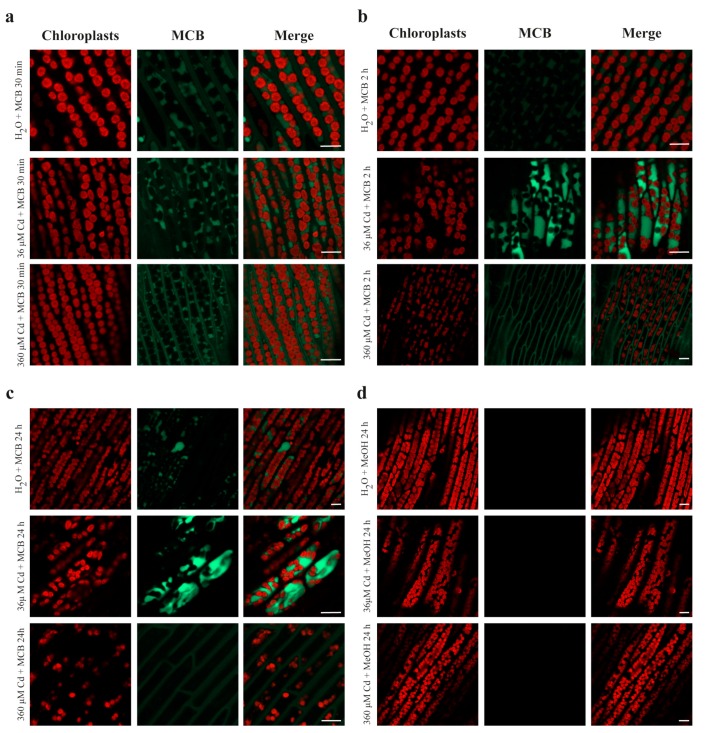
Confocal laser scanning microscopy (CLSM) imaging of *L. riparium* gametophytes (phylloids) exposed to 0 (Control), 36 and 360 µM CdCl_2_ for 7 days, followed by treatment with 100 µM MCB for 30 min, 2 h, and 24 h (green signal). Chlorophyll autofluorescence at the same exposure times is also visualized (red signal in chloroplasts), as well as the merge between MCB staining and chlorophyll autofluorescence. (**a**) MCB staining for 30 min. In control MCB-treated gametophytes, staining is visible in the cytosol and, partly, in the vacuoles. In samples treated with 36 µM CdCl_2_, MCB staining occurs in the cytosol and the vacuoles, whereas in the samples treated with 360 µM CdCl_2_, MCB fluorescence is predominantly present in the cytosol and much less in the vacuoles. (**b**) MCB staining for 2 h. Controls similar to (a). Differently, the 36-µM CdCl_2_ samples show strong MCB staining inside the vacuoles, whereas in the 360-µM CdCl_2_ samples the MCB signal is detected only in the cytosol. (**c**) MCB staining for 24 h. The overall situation is similar to (b) [(in controls also to (a)]. In addition, in the 360-µM CdCl_2_ samples, the chloroplasts are slightly dilated, possibly because of thylakoid membrane swelling, when compared to the round–shaped morphology of chloroplasts from the control samples (a). (**d**) Representative negative controls treated with methanol (MeOH) instead of MCB. All images were captured with a Zeiss LSM 800 CLSM at λ_EX_: 405 nm, λ_EM_: 490 nm for MCB (as for MeOH), and λ_EX_: 543 nm, λ_EM_: 608 nm for chlorophyll. Scale bars = 10 µm.

Control and Cd-treated *L. riparium* gametophytes (phylloids) were labeled *in situ* with 100 μM MCB for 30 min, 2 h, and 24 h (Figure 4). In controls, the mild MCB staining was localized in the cytosol and, partially, in the vacuoles, and remained at a fairly constant level at all exposure times (Figure 4a–c). In the 36 μM CdCl_2_-treated gametophytes, MCB-stained for 30 min, a fluorescent labeling in the cytosol and vacuoles, not too dissimilar from that of the controls, was observed (Figure 4a). In contrast, after 2 and 24 h, the MCB staining in the cytosol and vacuoles was much stronger than that in controls (Figure 4b,c). Concerning the 360 μM CdCl_2_ treatments, the MCB staining after a 30-min incubation was mainly visible in the cytosol and minimally in the vacuoles (Figure 4a), but after 2 and 24 h, it was localized only in the cytosol (Figure 4b,c). As negative control, incubation with methanol (instead of MCB) was carried out for each condition and treatment, to avoid erroneous interpretations of the fluorescence labels (Figure 4d).

Additionally, in accordance with the TEM observations (see below), the chlorophyll autofluorescence imaging of the 360 μM Cd-treated gametophytes (Figure 4c) revealed a slight dilatation of the chloroplasts, possibly due to the swelling of thylakoid membranes, in contrast to the round-shaped morphology of the chloroplasts in the control samples (Figure 4c).

### 2.4. Cd Treatments Caused Only Slight Cytohistological Damage to Gametophytes

**Figure 5:**
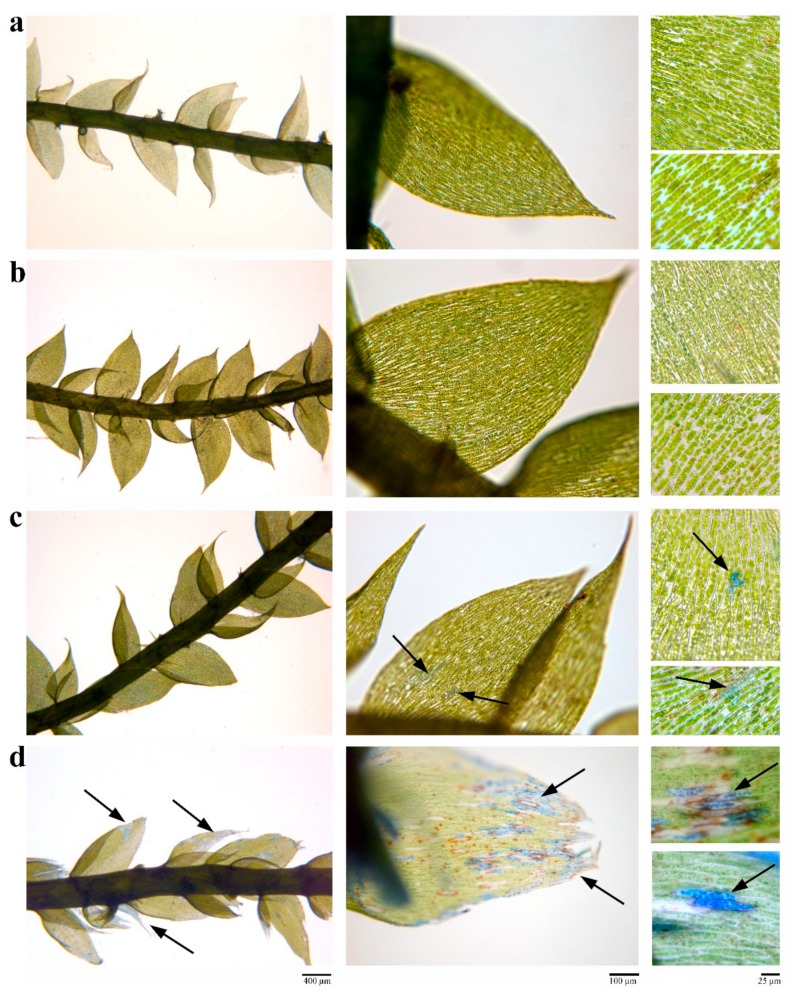
Evans Blue staining (2 h-treatment) of *L. riparium* gametophytes. (**a**) controls; (**b**) exposed to 36 μM Cd for 7 days; (**c**) exposed to 360 μM Cd for 7 days; (**d**) exposed to 100% ethanol for 1 h (positive controls). Arrows indicate cytohistological damage.

*L. riparium* gametophytes not exposed to Cd (controls) and stained with Evans Blue did not show any damage, particularly in phylloids (Figure 5a). Likewise, 36 and 360 μM Cd treatments did not show extensive damage (Figure 5b,c), but only slight injuries at the highest metal concentration (Figure 5c). By contrast, 1 h-exposure of gametophytes to pure ethanol (positive control) produced heavy alterations in the tissues (Figure 5d).

### 2.5. Cd Treatment Lowers Photosynthetic Activity in L. riparium Gametophytes

**Figure 6:**
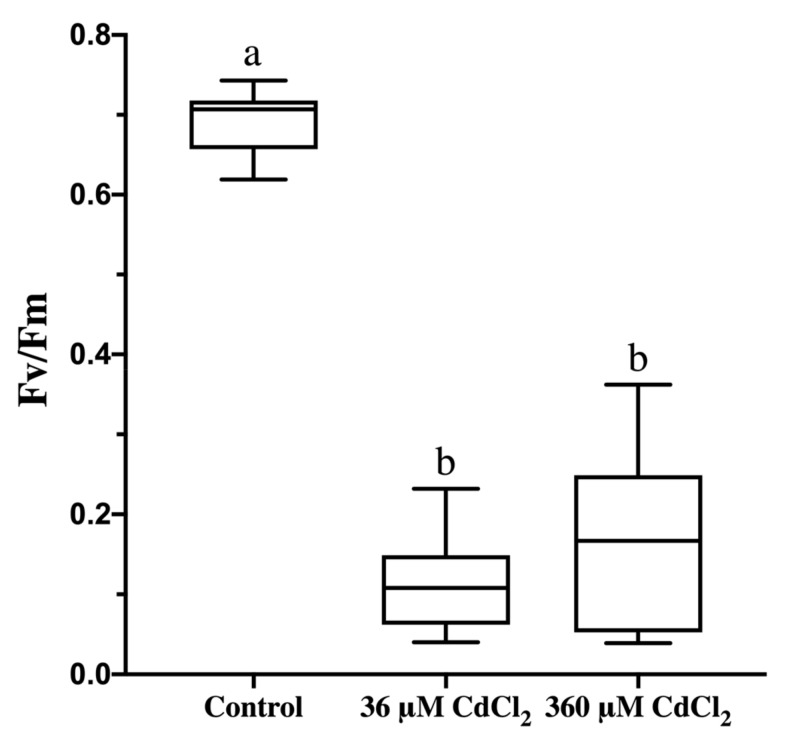
Photochemical efficiency (*Fv*/*Fm*) of *L. riparium* gametophytes exposed to 0 (Control), 36 and 360 μM CdCl_2_ for 7 days. Values are given by nine measurements *per* treatment, and expressed as mean ± SE; bars not accompanied by the same letter are significantly different at *p* < 0.05.

In order to evaluate the effects of Cd treatments on the photosynthetic activity in *L. riparium* gametophytes, the photochemical efficiency was assessed. Maximum PSII quantum yield (*Fv*/*Fm*) was negatively affected by both Cd concentrations (36 and 360 µM), compared to control (Figure 6).

### 2.6. Ultrastructural Observations Evidenced Slight Ultrastructure Alteration in Cd-Exposed Gametophytes

**Figure 7:**
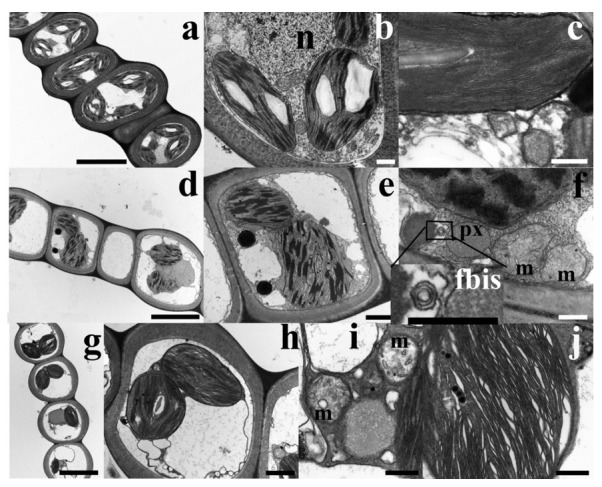
TEM micrographs of *L. riparium* gametophyte (phylloid) cells from samples exposed to 0 (Control) (a–c), 36 µM CdCl_2_ (d–f) and 360 µM CdCl_2_ (g–j) for 7 days. (**a**) Low-magnification micrograph of a gametophyte section. Each cell is characterized by a thick cell wall, lenticular chloroplasts in the peripheral cytoplasm with grana and intergrana thylakoids and starch grains inside, and a large central and electron-transparent vacuole. (**b**) Detail of a single cell delimited by a thick cell wall. Chloroplasts with grana and intergrana thylakoids and starch grains are visible, as well as a central nucleus (n) containing eu- and heterochromatin. (**c**) Detail of an unswollen chloroplast in which the thylakoids are arranged in tightly packed straight bands. (**d**) Low-magnification micrograph of samples treated with 36 µM Cd revealing an overall cell ultrastructure similar to the controls. (**e**) Micrograph of a single cell with misshaped chloroplasts and well-preserved grana and intergrana thylakoids and vacuoles. (**f**) Micrograph of two mitochondria (m) with a regular morphology, next to a peroxisome (px). (fbis) Detail of the area outlined in (**f**) showing a multilamellar body. (**g**) Low-magnification micrograph of samples treated with 360 µM Cd in which plasmolyzed cells delimited by thick cell walls, chloroplasts, and vacuoles are visible. (**h**) Detail of a single cell showing plasmolysis and a vacuolated cytoplasm still containing chloroplasts with grana and intergrana thylakoids and starch grains. (**i**) Detail of two mitochondria (m) with an electron-clear matrix and swollen cristae. (**j**) Detail of a chloroplast with diffuse thylakoid swelling. Scale bars = 5 µm (a, d, g), 1 µm (b, e, h), and 300 nm (c, f, fbis, i, j).

TEM micrographs of control (untreated) *L. riparium* gametophyte sections revealed that phylloid cells were surrounded by a thick cell wall, and contained several lenticular chloroplasts in the peripheral cytoplasm, as well as a central vacuole (Figure 7a). The well-developed thylakoids, arranged as grana and intergrana, were packed and tidily placed along the chloroplast main axis, without signs of swelling (Figure 7b,c); starch grains and rare plastoglobules were also visible (Figure 7b,c). Mitochondria had a typical morphology with cristae and an electron-dense matrix. Nuclei showed classical eu- and heterochromatin (Figure 7b). Conversely, in Cd-treated samples, some ultrastructural changes were observed that were more marked, albeit not severely, in the 360 µM Cd-treated phylloids. Samples exposed to 36 µM Cd had, in fact, a quite well-preserved ultrastructure, even though chloroplasts were slightly deformed (Figure 7d,e). The morphology of mitochondria was comparable to that of the controls (Figure 7f) and multilamellar bodies occurred in the cytoplasm (Figure 7fbis). Even more so, samples treated with 360 µM Cd had a number of ultrastructural alterations, such as plasmolyzed cells with some cytoplasm vacuolization (Figure 7g). Although grana and intergrana thylakoids were still present in the chloroplasts, a diffuse swelling was visible (Figure 7j). Mitochondria seemed altered, with swollen cristae and an electron-clear matrix. In some cells, precipitated electron-dense material was also present (Figure 7i).

## 3. Discussion

The moss *L. riparium* is able to detoxify (extremely) elevated concentrations of Cd (36 and 360 μM CdCl_2_) even when the metal is supplied for a prolonged time (7 days). The slight cytohistological and ultrastructural damage caused by Cd suggests very efficient metal detoxification processes functioning in this moss, despite that—as a general sign of suffering—photochemical efficiency was negatively affected by both Cd concentrations. Interestingly, the mechanisms based on Cd immobilization at the cell wall level have previously been demonstrated not to play a relevant role [31,34]. By contrast, intracellularly-synthesized stress proteins (such as the heat shock protein 70) might be important in repairing the damage caused by Cd, especially at high concentrations, possibly by allowing the correct refolding of Cd-impaired proteins [31,34].

Here, we found that to counteract (extremely) severe Cd stress, *L. riparium* gametophytes adopt a detoxification system employing, on the whole, thiol peptide compounds, such as γ-EC, GSH, and PCn. In particular, after 7 days of Cd treatment, PCn synthesis is induced only by the highest (360 μM Cd) and not by the lowest (36 μM Cd) metal concentration. This response demonstrates that PCn biosynthesis (in any case, minimally induced) is only triggered by an extremely high Cd concentration. Accordingly, the *in vitro* PCS activity measured in the gametophyte extracts reaches a *plateau* only after treatment with the highest Cd concentrations (100 and 360 μM Cd), whereas its activation is approximately half as high with the lowest concentration (36 μM Cd). Thus, especially in the presence of 36 μM Cd, other metal detoxification systems, rather than PCn, seem to operate effectively at an intracellular level.

In this regard, it should be pointed out that, at least in higher plants, the PCS enzyme does not possess an exclusive transpeptidase activity (i.e., a polymerase activity directed to PCn biosynthesis) [18], but also has a peptidase activity [21,22,23], because PCS belongs to the papain-like clan CA of the cysteine peptidases [35]. Thus, the “bifunctional” enzyme PCS can convert GSH to γ-EC by deglycination of GS-conjugates [21,22,23] and, consistently, contribute to the degradation of xenobiotics and/or metal-thiolate complexes in the cytosolic compartments. In this way, the high levels of GSH found in *L. riparium* gametophytes might be important catalytic promoters of the PCS activation toward the peptidase instead of the transpeptidase “direction”—even considering that Cd does not induce more PCn synthesis at 36 μM than in the controls, and induces only trace level PCn at 360 μM.

Indeed, unlike PCn, high GSH levels are detected both in the controls and in Cd-treated *L. riparium* gametophytes. Mosses are already known to be able to synthesize GSH at high levels, as shown by control and 36 μM Cd-exposed gametophytes of *Polytrichastrum formosum*, *Fontinalis antipyretica*, and *Hypnum cupressiforme*, in which up to ca. 370 nmol g^−1^ FW of GSH were measured [27]. Likewise, Bleuel et al. (2011) [36] detected about 200 nmol g^−1^ FW of GSH in the moss *Physcomitrella patens*. Indeed, GSH *per se* can represent an efficient system for Cd detoxification, particularly in bryophytes [37], but also in higher plants [7,38]. Moreover, besides their direct metal detoxification capacity, high levels of GSH are essential to neutralize ROS production, together with antioxidant enzymes, such as SOD and CAT. In our samples, these enzymes are activated by the two Cd concentrations, thus indicating that *L. riparium* owns an enzymatic arsenal that is collectively able to quench ROS even after 7 days of severe metal exposure.

Last but not least, GSH is also an essential co-substrate for GST activation. This enzyme, with cytosolic, chloroplastic, and nuclear isoforms in the moss *P. patens* [39], catalyzes the conjugation of GSH and, to a much lesser extent, of γ-EC [16,17] with several endogenous substances, xenobiotics, metals, etc. [10,11,12,13,39,40,41]. This conjugation is usually followed by vacuolar compartmentalization [39,42] and further intravacuolar degradation [43,44]. Interestingly, an hemerythrin class of GST that can bind metals, such as Fe and Cd [45], by means of a thiolate complex, has been discovered in *P. patens* [39]. In our experiments, in contrast to the PCS enzyme, the GST from gametophytes exposed to both Cd concentrations is much more active than in controls. Hence, GST can be activated, together with SOD and CAT, both to limit ROS production and to contribute to Cd detoxification by metal intravacuolar segregation. Consequently, the high levels of GSH found in *L. riparium* gametophytes might result, on the one hand, in a substrate for PCS activation in the cytosol toward the peptidase “direction” and, on the other hand, in a Cd detoxifying *per se*, as well as a co-substrate for GST, the activation of which can lead to vacuolar compartmentalization of the GS-conjugates.

The importance of the balance between cytosolic/vacuolar processes for Cd detoxification in *L. riparium* gametophytes, in particular in phylloids, is confirmed by the *in situ* labeling of the thiolic compounds with MCB at different time points (30 min, 2 h, and 24 h). After a 30-min treatment with MCB, in control and 36 μM Cd-exposed gametophytes, cytosolic and, in part, intravacuolar fluorescence is detected. After 2 and 24 h of MCB staining, a marked increase in cytosolic and, above all, intravacuolar fluorescence, is observed in the 36 μM Cd-exposed gametophytes, a possible sign of enhanced MCB staining due to the prolonged exposure to the dye. Accordingly, treatment of *P. patens* protonema cells with MCB led to labeling of the cytosol, followed by vacuolar internalization after 3 h of staining [36]. Moreover, the exposure of gametophytes to the highest concentration of Cd, deliberately supplied to burden the moss with an extremely severe metal stress, radically changes the scenario. Already after 30 min, and even after 2 and 24 h, the MCB fluorescence is evident only in the cytosol of the phylloid cells, but it is almost completely absent inside the vacuoles.

Altogether, under 36 μM Cd treatment, the PCS enzyme *in vitro* is more active than in the controls, but it is still much less active, by approximately 50%, than in the presence of the higher metal concentrations (100 and 360 μM Cd); above all, the PCn synthesized *in vivo* are present at levels not significantly higher than those in controls. Thus, under these conditions, the high GST activity, due to the high GSH levels, allows the vacuolar compartmentalization of Cd. In this process, ABC tonoplast transporters are possibly involved [14]. At the same time, the trend of PCS activation toward the peptidase “direction” may lead to some γ-EC production, possibly contributing to slight increase in the cytosolic MCB staining [16,17]. In fact, γ-EC, at least in *Arabidopsis thaliana*, cannot be considered a suitable substrate for ABC tonoplast transporters [14], and, hence, its intravacuolar fluorescence might be overlooked. In any case, with 36 μM Cd, the GST activity of *L. riparium* gametophytes seem to overcome the peptidase activity of the PCS.

Despite a PCn production higher in the 360 μM than in the 36 μM Cd-exposed gametophytes (and in controls), the PCn levels synthesized under this extremely high metal concentration are still very low, also when compared with those found in other bryophytes—specifically in *Sphagnum palustre* that, to our knowledge, is the only moss in which PCn were quantified and characterized [27]. Thus, in our samples, the direct contribution of PCn to Cd detoxification seems to be extremely limited. However, at the same time, at this Cd concentration, the PCS enzyme is fully active *in vitro*, having even reached a *plateau* in its activity. Thus, under this condition, PCS might reasonably be assumed to be mainly challenged for the cytosolic degradation of GS-Cd conjugates, i.e., the peptidase “direction”, rather than for the biosynthesis of PCn, i.e., the transpeptidase “direction”. Therefore, the MCB fluorescence constantly detected intracellularly might be a consequence of a PCS-dependent generation of the cleavage products in the cytosolic compartment. Indeed, when MCB is supplied together with Cd and other metals in *A. thaliana*, a significant amount of fluorescence is retained in the cytosol of the leaf cells [14]. Hence, a cytosolic formation of γ-EC-bimane may be postulated in this condition, through the Cd-triggered PCS activation, without or with a very low sequestration of this conjugate in the vacuolar compartment. All these processes suggest that PCS and GST may play a joint role in the intracellular detoxification of Cd, at least when the metal is supplied at extremely elevated concentrations and for a long time.

Thus, *L. riparium* seems to be an effective system for the study of Cd detoxification, also thanks to anatomical features that facilitate the metal uptake, such as lack of strong hydrophobic barriers and the absence of a vascular system *sensu proprio*. The main mechanisms underlying the high ability at counteracting the negative effects of (extremely) high Cd levels can be attributed to thiol peptide-mediated intracellular detoxification, as well as to activation of PCS and GST and, to some extent, to vacuolar compartmentalization. This study strengthens previous observations on the ability of *L. riparium* to tolerate strong metal pollution, clarifies its intracellular Cd detoxification mechanisms, and points to the potential use of this moss in biomonitoring and phytoremediation purposes.

## 4. Materials and Methods

### 4.1. Plant Material and Growth Conditions

Samples of *Leptodictyum riparium* (Hedw.) Warnst. (Bryophyta) were collected from a tap water-filled basin in the Botanical Garden of the University of Naples “Federico II” (Italy). Single gametophytes were carefully washed with deionized water, then surface-sterilized in 7% (*v*/*v*) NaClO with a few drops of Triton X-100, and thoroughly rinsed with deionized water. Samples were individually put into Falcon tubes filled with 45 mL of sterile tap water (control) or CdCl_2_ in two different concentrations (36 and 360 μM), for an overall metal exposure of 7 days. The cultures were placed in a growth chamber with night and day temperatures ranging, respectively, from 15 ± 1.3 °C to 20 ± 1.3 °C, 70% ± 4% relative humidity, a 16-h/8-h light/dark regime, and a photosynthetic photon flux density of 40 μmol m^−2^ s^−1^. To confirm the absence of damage due to the sterilization process, *L. riparium* gametophytes were observed every 2 days with a Leitz Aristoplan microscope (Leitz, Wetzlar, Germany) and a Wild Heerbrugg M3Z binocular (Leica, Nussloch, Germany). The plant material was grown in triplicate and all the experiments were repeated at least three times.

### 4.2. Detection of ROS Production and SOD, CAT, and GST Activities

A spectrofluorometric assay employing 2′,7′-dichlorofluorescein diacetate (DCFH–DA) was performed for measurements of ROS production; the assay is based on intracellular de-esterification of DCFH-DA and its conversion to nonfluorescent 2′,7′-dichlorofluorescein (DCFH), which is then oxidized by ROS to the highly fluorescent 2′,7′-dichlorofluorescein (DCF) [46]. Moss samples were immediately frozen in liquid nitrogen and ground thoroughly with prechilled mortar and pestle. The resulting powder (150 mg) was then resuspended in Tris HCl 40 mM pH 7.4, sonicated, and centrifuged at 12,000× *g* for 30 min. The supernatant (500 μL) was collected, and protein content determined according to the Bradford’s method [47]. An aliquot (10 μL) of each sample was transferred to a 96-well plate, incubated with 5 μM DCFH-DA for 30 min at 37 ± 1 °C, and analyzed with an automatic plate reader. ROS amounts were monitored by fluorescence (excitation wavelength of 530 nm and emission wavelength of 660 nm).

One gram of moss gametophytes was ground with 1 mL of chilled NaH_2_PO_4_/Na_2_HPO_4_ buffer (PBS, 50 mM, pH 7.8) containing 0.1 mM ethylenediaminetetraacetic acid (EDTA) and 1% (*w*/*v*) polyvinylpyrrolidone (PVP). The homogenate was centrifuged at 12,000× *g* for 30 min, and the supernatant (enzyme extract) was collected for protein quantification and determination of SOD, CAT, and GST activities. The protein concentration was quantified spectrophotometrically at 595 nm according to the Bradford method with bovine serum albumin (BSA) as the standard [47].

CAT activities were calculated and expressed as the absorbance decreased at 240 nm due to H_2_O_2_ consumption with a commercial kit (Sigma-Aldrich, St. Louis, MO, USA), according to the manufacturer’s protocol. SOD activity was spectrophotometrically determined at 450 nm with a commercial kit (19160, Sigma-Aldrich). The assay utilizes a water-soluble tetrazolium salt that produces a formazan dye after reduction by the radical anion superoxide (•O_2_^–^). The reduction rate with •O_2_^–^ is linearly related to the xanthine oxidase activity, which is inhibited by SOD. The result was compared with a standard SOD curve. One unit of SOD activity was defined as the amount of enzyme that inhibited 50% of the •O_2_^–^ reduction per min at 25 °C and pH 7. GST activity was measured with a commercial kit (CS0410, Sigma-Aldrich). The GST-catalyzed conjugation of GSH to 1-chloro-2,4-dinitrobenzene (CDNB) was monitored at 340 nm for 4 min. The reaction mixture contained 4 μL of extract and 196 μL of reaction solution (200 mM GSH and 100 mM CDNB in Dulbecco’s buffer at pH 7). A GST unit was defined as the amount of enzyme that catalyzes the formation of 1 μmol of the GS-DNB conjugate per min at 25 °C and pH 7 (ε = 9.6 mM^−1^ cm^−1^ according to Habig and Jakoby 1981 [48]).

### 4.3. In Vitro Activity Assay of PCS

*L. riparium* PCS activity was assayed in extracts of fresh Cd-untreated gametophytes (200 mg), as described in Petraglia et al. (2014) and Wojas et al. (2008) [27,49], with some modifications. Briefly, each gametophyte sample was frozen with liquid nitrogen in a 2-mL Eppendorf tube containing 2 agate grinding balls (5 mm diameter); each tube was then placed in a mixer mill (MM200, Retsch, Haan, Germany) and shaken with a frequency of 30 Hz for 1 min. The powder obtained was added with 600 μL of extraction buffer (20 mM HEPES-NaOH pH 7.5; 10 mM β-mercaptoethanol; 20% (*w*/*v*) glycerol; 100 mg mL^−1^ polyvinylpyrrolidone) and homogenized for 1 min with another cycle of mixer mill shaking. The homogenized samples were then centrifuged twice at 13,000× *g* (Hermle, Z 300 K, Wehingen, Germany) at 4 °C for 10 min; an aliquot of 400 μL of the supernatants was mixed with 100 μL of reaction buffer (250 mM HEPES-NaOH pH 8.0; 10% (*w*/*v*) glycerol; 25 mM GSH). The extraction and reaction buffers contained 36, 100, and 360 μM Cd supplied as CdCl_2_. After an incubation time of 90 min at 35 °C, the reaction was terminated with 125 μL 20% (*w*/*v*) trichloroacetic acid. The PCS activity was immediately assayed by HPLC–ESI–MS–MS and expressed as pmol PCn g^−1^ FW min^−1^.

### 4.4. γ–EC, GSH, and PCn Extraction, Characterization, and Quantification

On the 7th day of growth, single gametophytes were carefully washed with deionized water and gently blotted dry with filter paper. Then, 100 mg of each sample were put into a 2-mL Eppendorf tube, briefly frozen in liquid nitrogen, and stored in the dark at −80 °C until further analysis.

Each sample was extracted as described in Bellini et al. (2019) [50]. Briefly, gametophytes were homogenized by a mixer mill (MM200, Retsch) with 2 agate grinding balls (5 mm diameter) at a vibrational frequency of 30 Hz for 2 min. Then, 300 µL of ice-cold extraction buffer (5% (*w*/*v*) 5-sulfosalicylic acid (SSA), 6.3 mM diethylenetriamine-pentaacetic acid (DTPA), and 2 mM Tris (2-carboxyethyl) phosphine (TCEP)) were added to each homogenate, together with ^13^C_2_,^15^N-GSH, and ^13^C_2_,^15^N-PC_2_ as internal standards (each at a concentration of 200 ng mL^−1^). The powder was resuspended, kept in an ice bath for 15 min, and vortexed each 5 min. The extract was sedimented by centrifugation at 10,000× *g* (Z 300 K; Hermle, Wehingen, Germany) at 4 °C for 20 min. Each supernatant was filtered by a Minisart RC4 0.45-µm filter (Sartorius, Goettingen, Germany) and samples were stored at −80 °C until analysis.

Thiol peptides (γ-EC, GSH, and PCn) were analyzed with an 1290 Infinity UHPLC (Agilent, Santa Clara, CA, USA), equipped with a thermostated autosampler, a binary pump, and a column oven, coupled to an API 4000 triple quadrupole mass spectrometer (AB Sciex, Concord, ON, Canada), equipped with a Turbo-V ion spray source (AB Sciex). Chromatographs were separated by a reverse-phase Phenomenex (Torrance, CA, USA) Kinetex 2.6 µm XB-C18 100 Å, 100 × 3 mm HPLC column, protected by a C18 3-mm ID security guard ULTRA cartridge, as described in Bellini et al. (2019) [50]. The separation was achieved by means of a gradient solvent system (solvent A, acetonitrile with 0.1% (*v*/*v*) formic acid; solvent B, water with 0.1% (*v*/*v*) formic acid) as follows: solvent A was set at 2% for 5 min, raised with a linear gradient to 44% in 4.5 min, and then raised with a linear gradient to 95% in 1 min. Solvent A was maintained at 95% for 1 min before column re-equilibration (2.5 min). Flow rate and column oven temperature were set to 300 μL min^−1^ and 30 °C, respectively. The injection volume was 20 μL. Thiol peptides were identified and quantified by tandem mass spectrometry (MS/MS) with certified standards (GSH, PC_2–4_; AnaSpec Inc., Fremont, CA, USA) to build external calibration curves and certified glycine-^13^C_2_,^15^N-labeled GSH (Sigma-Aldrich) and glycine-^13^C_2_,^15^N-labelled PC_2_ (AnaSpec Inc.) as internal standards. System control, data acquisition, and processing were carried out by an Analyst^®^ version 1.6.3 software (AB Schiex, Concord, ON, Canada). The method was validated as described in Bellini et al. (2019) [50].

### 4.5. Confocal Laser Imaging of MCB Internalization

At least three samples of *L. riparium* gametophytes for each Cd treatment (36 and 360 μM CdCl_2_) and control condition were treated on a rocking shaker with 100 μM MCB (Thermo Fisher Scientific, MA, USA) for 30 min, 2 h, and 24 h at 21 °C in the dark, at near-neutral pH conditions.

Phylloids from gametophytes were washed in sterile water and observed with a Zeiss 800 confocal laser scanning microscope (CLSM) using a 63× immersion objective. For the detection of MCB and chlorophyll fluorescence, excitation was set at 405 and 543 nm, and emission was captured at 490 and 608 nm, respectively. MCB stock solutions were prepared at a 50 mM concentration in methanol, stored at −20 °C, and thawed immediately prior to use, with subsequent dilution up to 100 μM by adding sterile water. As control of the MCB staining, the same amount of methanol used for the 100 μM MCB treatments was added to the growth medium of the unstained *L. riparium* gametophytes.

### 4.6. Evans Blue Staining and Microscopy

Viability assay was performed using Evans Blue staining in order to detect cell damage/death as described in de León et al. (2007) [51]. At least three samples of *L. riparium* gametophytes for each growth condition (0, 36 and 360 μM CdCl_2_) and three positive controls (100% ethanol for 1 h) were incubated for 2 h with 0.05% Evans Blue, and then washed 4 times with deionized water to remove excess dye. Material was then mounted on a slide in 100% glycerol and examined for Evans Blue staining using light microscope (Leitz, Wetzlar, Germany) equipped with a Leica DFC 420 camera (Leica, Nussloch, Germany). 

### 4.7. Photochemical Efficiency

Maximum quantum yield of PSII (*Fv*/*Fm*) was measured by a chlorophyll fluorometer (Handy PEA, Hansatech Instruments, Ltd., UK) at 20 ± 1.3 °C temperature. Gametophytes were covered with a leaf clip to adapt them to darkness for 30 min and then exposed for 1 s to 3500 µmol photons m^−2^ s^−1^ (650 nm peak wavelength) and chlorophyll *a* fluorescence was recorded. Nine measurements were taken for each treatment and the fluorescence data were processed by PEA plus software (Hansatech Instruments, Pentney, King’s Lynn, UK).

### 4.8. Ultrastructural Observations

Gametophytes were fixed in 3% (*v*/*v*) glutaraldehyde in phosphate buffer solution (pH 7.2–7.4) for 2 h at room temperature, post-fixed with buffered 1% (*w*/*v*) OsO_4_ for 1.5 h at room temperature, dehydrated with ethanol up to propylene oxide, and embedded in Spurr’s epoxy medium [52]. Ultra-thin (40-nm thick) sections of gametophyte phylloids were put on 300-mesh Cu grids, stained with Uranyl Replacement Stain UAR (Electron Microscopy Science, Hatfield, PA, USA) and lead citrate, and observed under a Philips EM 208S TEM [52]. Fifty-four specimens were observed, with each set made up of three specimens collected twice and in triplicate from different dishes.

### 4.9. Statistical Analysis

Data were analyzed by means of the Graph-Pad Prism 8.2.1 statistical program (GraphPad Software Inc., San Diego, CA, USA). Data were reported as the mean ± SE (standard error). The threshold of statistical significance was set at *p* < 0.05, unless otherwise specified. The effect of Cd concentrations in terms of ROS production, SOD, CAT, GST, and PCS activities, were examined by one-way analysis of variance (ANOVA), followed by Tukey’s multiple comparison post-hoc test. Moreover, the data relating to PCn *in vivo* production were analyzed by two-way ANOVA, followed by Tukey’s post–hoc test as above.

## Supplementary Materials

The following are available online at https://www.mdpi.com/1422-0067/21/5/1583/s1. Figure S1; Table S1. Figure S1. Representative Selective Reaction Monitoring (SRM) chromatograms of L. riparium gametophytes treated with 360 mM CdCl2 for 7 days in the time range of 0–10 min runs. γ-EC and GSH were diluted 1:100 before the HPLC-ESI-MS-MS analysis. Three transitions (represented with different colors) were monitored per each analyte: based on signal to noise ratio, one of them was used as quantifier and the other two as qualifiers. Asterisk indicates stable isotope-labelled internal standard. Table S1. ROS quantification and antioxidant/detoxifying enzyme activities in L. riparium gametophytes treated with 0 (Control), 36 or 360 μM CdCl2 for 7 days. Values are mean ± SE.

## Abbreviations

PCn	Phytochelatins
PCS	Phytochelatin synthase
GSH	Glutathione
GS–bimane	Glutathione–bimane
γ–EC	γ–glutamylcysteine
ROS	Reactive oxygen species
SOD	Superoxide dismutase
CAT	Catalase
GST	Glutathione-*S*–transferase
MCB	Monochlorobimane
