# Supplementary material for: The Moss Leptodictyum riparium Counteracts Severe Cadmium Stress by Activation of Glutathione Transferase and Phytochelatin Synthase, but Slightly by Phytochelatins
Source: Int J Mol Sci. 2020 Feb 26;21(5):1583. doi: 10.3390/ijms21051583 (PMC7084805; doi:10.3390/ijms21051583)
Supplement: Supplementary file 1 [file ijms-21-01583-s001.pdf]

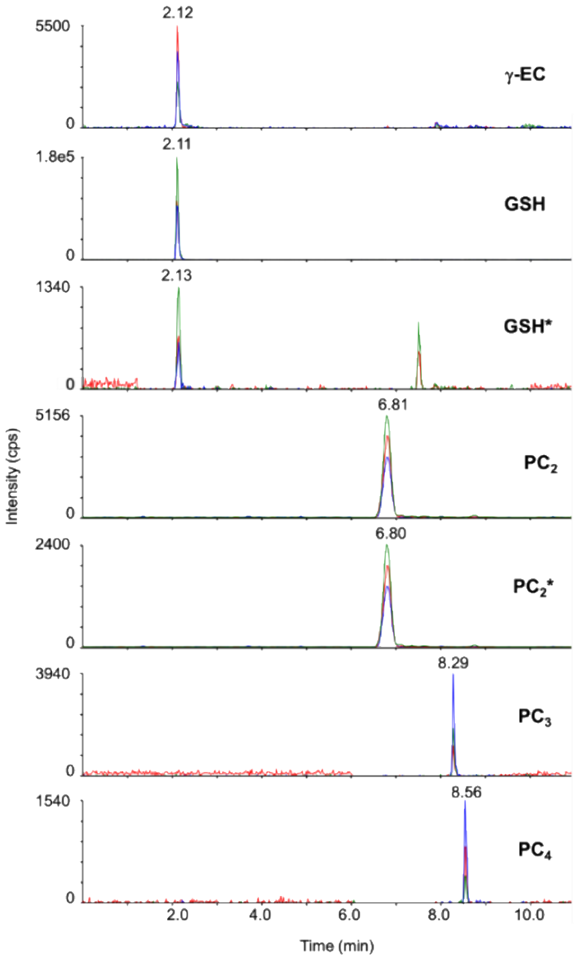

Figure S1. Representative Selective Reaction Monitoring (SRM) chromatograms of L. riparium gametophytes treated with 360 μM $CdCl_2$ for 7 days in the time range of 0-10 min runs. γ-EC and GSH were diluted 1:100 before the HPLC-MS-MS analysis. Three transitions (represented with different colors) were monitored per each analyte: based on signal to noise ratio, one of them was used as quantifier and the other two as qualifiers. Asterisk indicates stable isotope-labelled internal standard.

Table S1. ROS production and antioxidant/detoxifying enzyme activities in L. riparium gametophytes treated with 0 (Control), 36 μM or 360 μM $CdCl_2$ for 7 days. Values are mean ± SE.

	**Control**	**36 μM $CdCl_2$**	**360 μM $CdCl_2$**
**ROS (Fluorescence intensity)**	260.070 ± 20.180	1977.011 ± 25.051	2580.460 ± 109.196
**SOD activity (%)**	20.873 ± 1.564	57.621 ± 0.909	82.915 ± 1.093
**CAT activity (U mg^{-1})**	12.891 ± 0.578	43.412 ± 0.888	149.693 ± 0.705
**GST activity (μmol ml^{-1} min^{-1})**	0.558 ± 0.074	1.519 ± 0.040	1.972 ± 0.016
